# A pediatric emergency prediction model using natural language process in the pediatric emergency department

**DOI:** 10.1038/s41598-025-87161-x

**Published:** 2025-01-28

**Authors:** Arum Choi, Chohee Kim, Jisu Ryoo, Jangyeong Jeon, Sangyeon Cho, Dongjoon Lee, Junyeong Kim, Changhee Lee, Woori Bae

**Affiliations:** 1 Department of Radiology, College of Medicine, The Catholic University of Korea, Seoul, Korea; 2 VUNO, Seoul, Korea; 3 Department of Emergency Medicine, Seoul St. Mary’s Hospital, College of Medicine, The Catholic University of Korea, Seoul, Korea; 4 Department of Artificial Intelligence, Chung-Ang University, Seoul, Korea; 5 Department of Artificial Intelligence, Korea University, Seoul, Korea

**Keywords:** Pediatric emergency department, Emergency room visits, Prediction model, Natural language process, Language model, Paediatrics, Computer science

## Abstract

This study developed a predictive model using deep learning (DL) and natural language processing (NLP) to identify emergency cases in pediatric emergency departments. It analyzed 87,759 pediatric cases from a South Korean tertiary hospital (2012–2021) using electronic medical records. Various NLP models, including four machine learning (ML) models with Term Frequency-Inverse Document Frequency (TF-IDF) and two DL models based on the KM-BERT framework, were trained to differentiate emergency cases using clinician transcripts. Gradient Boosting, among the ML models, performed best with an AUROC of 0.715, AUPRC of 0.778, and F1-score of 0.677. DL models, especially the fine-tuned KM-BERT model, showed superior performance, achieving an AUROC of 0.839, AUPRC of 0.879, and F1-score of 0.773. Shapley-based explanations provided insights into model predictions, underlining the potential of these technologies in medical decision-making. This study demonstrates the potential of advanced DL techniques for NLP in emergency medical settings, offering a more precise and efficient approach to managing healthcare resources and improving patient outcomes.

**Supplementary Information:**

The online version contains supplementary material available at 10.1038/s41598-025-87161-x.

## Introduction

### Emergency department overcrowding in Korea

Emergency departments (EDs) worldwide, including Korea, are facing the problem of overcrowding, resulting in the overloading of medical staff and inefficient use of ED resources^1–3^. Overcrowding in EDs can lead to negative outcomes such as decreased quality of care, patient safety concerns, and higher healthcare costs^4^. ED overcrowding is a serious problem, especially in pediatric EDs (PEDs), where timely and accurate treatment is important^5^. The challenge of accurately diagnosing and treating children, who are less able to communicate their symptoms than adults, is exacerbated in overcrowded and resource-constrained settings^6^.

### Unnecessary PED visits

PED visits among pediatric patients continue to increase, and a larger proportion of these patients have repeated visits to the PED than adults. While adults often visit the ED for chronic disease issues, children who are more susceptible to infections tend to visit the ED more frequently the younger they are^7^. The high rate of unnecessary, unplanned PED visits in children not only contributes to PED overcrowding but may also reflect poor quality of care^8,9^. Addressing these challenges is critical to improving children’s health outcomes and optimizing emergency health care.

### Advantages of application of natural language process using electronic medical records

In the ED, it is crucial to accurately assess a patient’s condition and determine appropriate treatment priorities. Typically, prediction models in the ED are built using patients’ lab results or numerical data^10,11^. However, before structured patient lab results are available, clinicians describe information about a patient’s condition and symptoms in the electronic medical record (EMR) in an unstructured form in a natural language. Traditional ML methods are limited by their reliance on structured data, such as detailed (well-organized) patient descriptions and lab results. Obtaining this type of data is often a slow and resource-intensive process that demands significant clinical expertise. Applying cutting-edge natural language processing (NLP) techniques based on deep learning (DL) models to these clinician transcripts can help clinicians make real-time decisions, furnishing personalized, evidence-based recommendations that consider each patient’s history and symptoms^12^. Using this technology, the triage process in the ED can be optimized by assessing the severity and immediacy of patient conditions, thereby improving resource allocation. This leads to shorter wait times and enhances satisfaction for patients and healthcare staff, ensuring a more effective and efficient emergency care delivery system^13,14^. Despite their widespread use and clear benefits, the complete utilization of DL-based NLP method in clinical settings is limited by the lack of annotated data and automated tools that are essential for effectively extracting clinical insights^15^.

### Purpose

Overcrowding in pediatric emergency departments is often caused by unnecessary visits, leading to the inefficient use of resources and operational inefficiencies. This study aims to develop a DL-based NLP triage model for pediatric emergency patients to address this issue.

## Materials and methods

### Study population

**Fig. 1:**
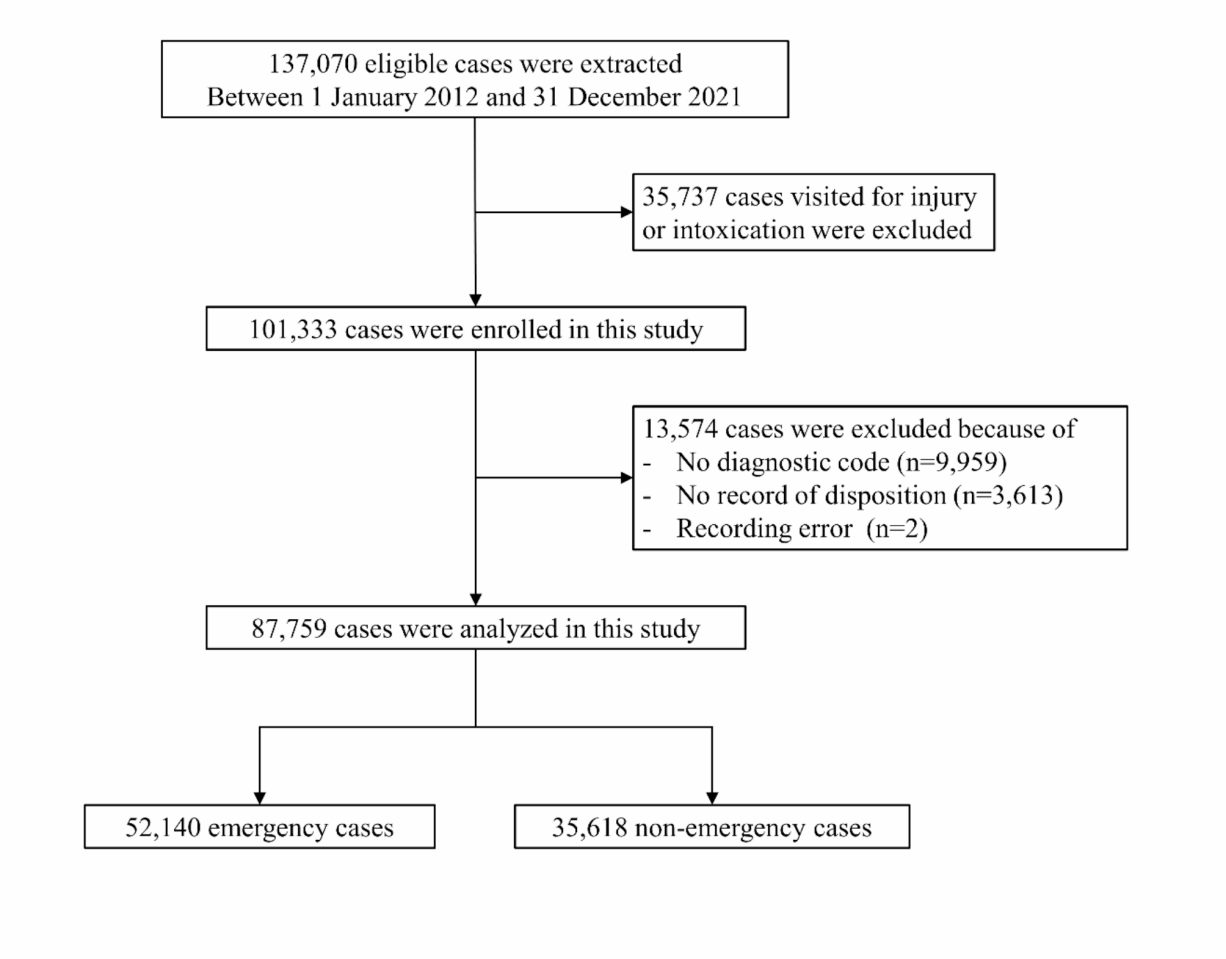
Flowchart of the study population.

This retrospective cohort study used EMR from the PED in a tertiary hospital in South Korea. We accessed the EMR on April 21, 2023, and information that could identify individual participants has been anonymized. The study participants were patients aged < 18 years who visited the PEDs between January 1, 2012, and December 31, 2021. The cohort was formed by excluding those who visited the PED for the treatment of injury or intoxication, missing a diagnosis code, visited the PED for purposes other than medical treatment, or had no record of disposition. The final cohort of interest included 87,759 patients, including 52,140 emergency patients (Fig. 1.).

### Emergency and non-emergency cases

Traditional triage systems such as the Canadian Triage and Acuity Scale (CTAS) or the Korean Triage and Acuity Scale (KTAS) classify the urgency of emergency department patients from level 1 (critical) to level 5 (non-urgent) based on their symptoms and vital signs. This classification determines treatment priority, with levels 1 through 3 typically considered emergency cases requiring immediate attention, while patients classified as levels 4 or 5 are considered non-emergency cases and may be treated with lower priority.

In this study, we developed a novel approach to classify emergency and non-emergency cases in the PED, addressing limitations in traditional triage systems. While conventional methods often rely on initial triage scores, such as the CTAS or KTAS, these can lead to over-triage or under-triage of patients^16,17^. In actual PED, many patients at triage levels 1, 2, and 3 are discharged without receiving emergency treatment, while some patients at lower triage levels still require urgent care. To address the limitations of this triage system, we defined patients who received emergency treatment as the “emergency group” and those discharged without such treatment as the “non-emergency group,” based on whether they received emergency care during their visit.

“Emergency” was defined as “a sudden, usually unexpected event that requires immediate action to minimize adverse consequences”^18^. Emergency cases included those who underwent blood tests, urinalysis, intravenous hydration, nebulization, immediate administration of medication in the PED, and those who were hospitalized. A detailed distribution of emergency cases according to intervention criteria is provided in Supplementary Fig. 1. “Non-emergency” refers to a clinical condition that does not require immediate medical attention, diagnosis, or treatment. Non-emergency cases included those who were discharged from the PED without immediate testing or medication or were discharged with only discharge medication.

### Preprocessing of unstructured clinical transcript

The unstructured data consists of clinicians’ written descriptions, transcribed during a patient’s visit to the ED, detailing their condition, chief complaints, and symptoms. The clinician transcripts used in our study were written without a standardized form, with medical terms presented in Korean or English and with numeric values and special characters. To enhance the effectiveness of handling clinician transcripts, we systematically separated Korean, English, numbers, and special characters before incorporating text information into the prediction models. For instance, if Korean characters are followed by English, numbers, or special characters in a clinician transcript, we insert a blank space to ensure a clear distinction among Korean, English, numbers, and special characters.

### Prediction models

**Fig. 2:**
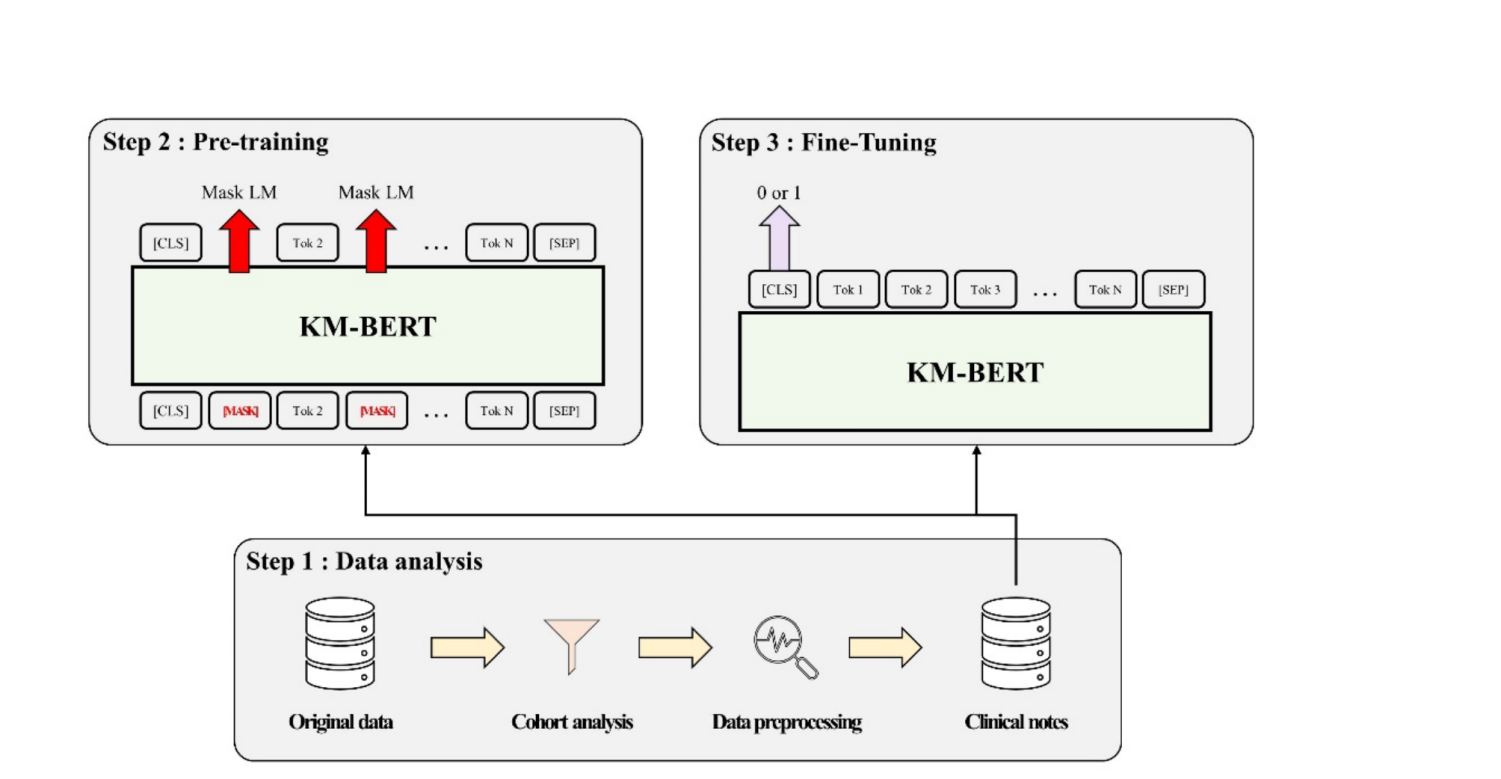
Overview of the study process from data extraction to model evaluation.

Figure 2 showed a visual overview of the entire process from data extraction to model training and evaluation.

#### Machine learning (ML)-based prediction model using TF-IDF

Term frequency-inverse document frequency (TF–IDF) is a traditional NLP method that serves as a statistical measure that assesses the relevance of a word to a document within a collection of documents. This is determined by multiplying two metrics: the TF score, which denotes how frequently a word appears within a document, and the IDF score, which indicates the rarity of a word across an entire set of documents. A word was considered more significant when assigned a higher TF-IDF value. In this study, we applied TF-IDF by treating the triage note for each patient as a document. Four ML models—one statistical method (logistic regression) and three tree-based ensemble methods (random forest, gradient boosting, and XGBoost)—were trained to predict emergency and non-emergency cases by treating the TF-IDF scores applied to the clinician transcripts as input features.

#### Introduction to deep learning-based NLP methods

Word2Vec is a traditional DL-based NLP method that was developed to map words into an embedded space and captures their semantic meanings by positioning words with similar meanings close to each other^19^. Methods based on Word2Vec have been empirically validated as useful for analyzing the relationships between medical terms and symptoms^20^. For example, they can help identify connections between various diagnostic terms for a specific symptom or explore associations between different treatments.

Long short-term memory (LSTM) is a type of DNN specifically designed to process sequential data. It has been widely applied to numerous NLP tasks because of its ability to extract semantically useful information from entire sentences^21^.

#### Deep learning-based prediction model using KM-BERT

We derived our DL-based prediction model using KM-BERT, a Korean language model pretrained on a collection of three types of Korean medical documents: medical textbooks, health information news, and medical research articles. In particular, we employed KM-BERT with a small vocabulary comprising 16,424 subwords. We further pretrained it on our clinician transcripts using the pretext task of masked language model (MLM). Our pretraining encourages the model to learn the semantic context by randomly masking subwords in a sentence and reconstructing the missing subwords based on the context provided by the remaining subwords. We then fine-tuned the KM-BERT model on our clinician transcripts to predict emergency and non-emergency cases, using the subwords of the clinician transcripts as input sequence. This prediction model is denoted as KM-BERT (MLM).

### Model training and testing

The cohort was randomly divided (64:16:20) into training, validation, and testing sets using the Python (version 3.9.13) package *scikit-learn* (version 1.2.1). The same data split was used to train and evaluate the ML-based and DL-based prediction models. For model evaluation, we used bootstrapping on the testing set with a sampling ratio of 0.75, conducting 10 random iterations to produce results with mean and standard deviations. The discriminative power of the prediction models was assessed using the following key metrics: area under the receiver operating characteristic curve (AUROC), area under the precision-recall curve (AUPRC), and F1-score. The AUROC represents the probability that a randomly selected patient who was emergent was assigned a higher risk than a patient who was not emergent. The AUPRC is a critical metric for problems where properly classifying the positives (in our study, the emergent cases) is important. Meanwhile, the F1-score is a harmonic mean of precision (i.e., the ratio of true positives among samples with predicted positive labels) and recall (i.e., the ratio of true positives among samples with ground-truth positive labels).

To train the ML-based prediction models, we first constructed a medical dictionary to extract medical terms from the corpus of our clinician transcripts using medical dictionaries and aggregated Korean and English words with the same clinical meaning. We identified the most frequent words in the entire dataset by calculating the cumulative sum, which accounted for 85% of the overall word distribution across the clinician transcripts. This yielded a final count of 103. We then applied TF-IDF using the Python package *scikit-learn* and trained the following ML prediction models using the TF-IDF scores on the most frequent words as input features: logistic regression, random forest, and gradient boosting using the Python package *scikit-learn* and XGBoost using the Python package *xgboost* (version 1.7.6). The hyperparameters of the ML models were chosen via a grid search, that is, max depth from the candidate values {1,2,3,4,5}, the number of estimators from {100, 200, 300, 400, 500} for the tree-based ensemble models, and the regularization coefficient from {0.001, 0.01, 0.1, 1, 10, 100, 1000} for logistic regression, using the AUROC performance on the validation set.

To train the DL-based prediction model using KM-BERT, we initially pretrained KM-BERT with a small vocabulary by employing an MLM pretext task on the training set. Specifically, we processed each clinician’s transcript using the subword tokenizer available in KM-BERT, utilizing the resulting subword tokens as input features. Subsequently, we added a binary classification layer consisting of one fully connected layer on top of the [CLS] token from the pretrained KM-BERT. We fine-tuned the prediction model to classify emergency and non-emergency cases. Pretraining and fine-tuning incorporated early stopping to select the model with the lowest validation loss using a batch size of 64. The learning rate was set to 5e-4 during pretraining, and for fine-tuning, it was set to 1e-5 and 1e-2 for KM-BERT and the classifier, respectively.

### SHAP-based rationalization

DL models have improved the performance of NLP tasks; however, their complexity makes it difficult to understand the model output. To bridge this gap, various explainability techniques have been suggested to elucidate model predictions; rationalization is a type of explainability method that provides natural language explanations^22^. In this work, we applied Shapley-based rationalization to provide extractive rationales, which provide words that explain why the model makes a specific prediction^23^. We computed the attribution of the individual words by assessing the variance in the model output when a word was included versus when it was omitted. To increase interpretability, we intentionally computed the attribution of words instead of tokens. For instance, if the model prediction is an emergent case and the word “fever” has positive attribution, this indicates that “fever” contributed to the model in making the prediction an emergent case.

### Subgroup analysis

The Korean Triage and Acuity Scale (KTAS) is a Korean emergency patient classification tool that evaluates the severity and urgency of a patient’s condition based on their symptoms, categorizing them into five levels to determine the priority of treatment^11^. The analysis targeted pediatric patients with KTAS levels from 2016 to 2021. KTAS levels 1–3 was classified as emergency, levels 4–5 were classified as non-emergency, and their performances were compared with other models.

### Ethics statement

This study was approved by the Institutional Review Board (IRB) at The Catholic University of Korea (IRB approval number KC23RISI0073). The Institutional Review Board (IRB) at The Catholic University of Korea waived the requirement for informed consent. All procedures were conducted in compliance with applicable guidelines and regulations.

## Results

### Performance of pediatric emergency prediction models

**Fig. 3:**
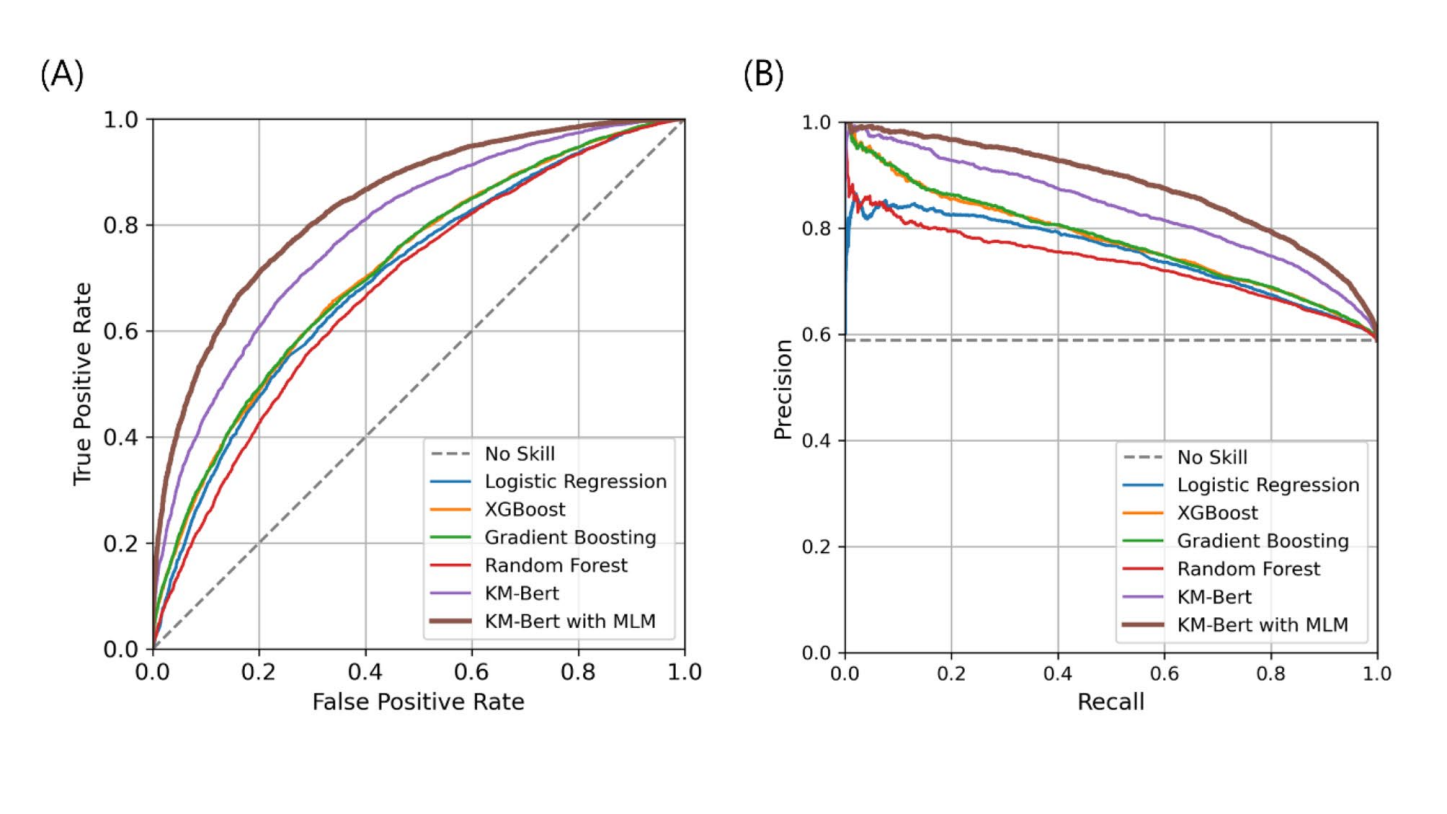
Prediction Ability of ML-based and DL-based NLP Techniques for Pediatric Emergency Prediction. (A) Receiver operating characteristic curves. (B) Precision-recall curve. The corresponding values of the area under the curve for each model are shown in Table 1. KM-BERT, Korean medical bidirectional encoder representations from transformers; MLM, masked language modeling; AUROC, area under the receiver operating characteristics; AUPRC, area under the precision-recall curve.

The average length of the clinicians’ transcripts was 62.5 words (SD = 36.7). We trained four topic models with TF-IDF utilizing statistical and ensemble ML models, including logistic regression, XGBoost, gradient boosting, and random forest, and two DL-based language models based on KM-BERT to predict emergent cases using clinician transcripts as input. The top-ranked words extracted by TF-IDF and their corresponding scores are presented in Appendix Table A1 and visually represented in Appendix Figure A1, providing insight into the key terms identified by our ML model. The performance results for each model are presented in Table 1. and Fig. 3. Gradient boosting shows the highest AUROC of 0.715 ± 0.002, AUPRC of 0.778 ± 0.001, and recall of 0.626 ± 0.003, while XGBoost leads to precision of 0.741 ± 0.001. Gradient boosting also achieved the highest F1-score of 0.677 ± 0.001 and accuracy of 0.649 ± 0.001. Gradient boosting and XGBoost had the lowest Brier scores (0.209 ± 0.002), indicating the most accurate probabilistic predictions. However, the two DL-based prediction models exhibited better performance than the ML-based models with TF-IDF. Comparing the two DL-based NLP methods, the fine-tuned KM-BERT with MLM outperformed the KM-BERT for all indicators. In AUROC, it is 0.839 ± 0.001, which is higher than that of KM-BERT (0.788 ± 0.002), and in AUPRC, it is 0.879 ± 0.001, which is higher than that of KM-BERT (0.837 ± 0.002). In recall, it is 0.724 ± 0.002, slightly higher than that of KM-BERT (0.719 ± 0.002), while in precision, it is 0.828 ± 0.002, which is a clear difference from that of KM-BERT (0.775 ± 0.002). The F1-score is 0.773 ± 0.001, which is higher than that of KM-BERT (0.746 ± 0.002), and the accuracy is 0.749 ± 0.002, which is higher than that of KM-BERT (0.712 ± 0.002). KM-BERT with MLM has a Brier score of 0.164 ± 0.001, which is lower than that of KM-BERT (0.188 ± 0.001).

### Calibration

**Fig. 4:**
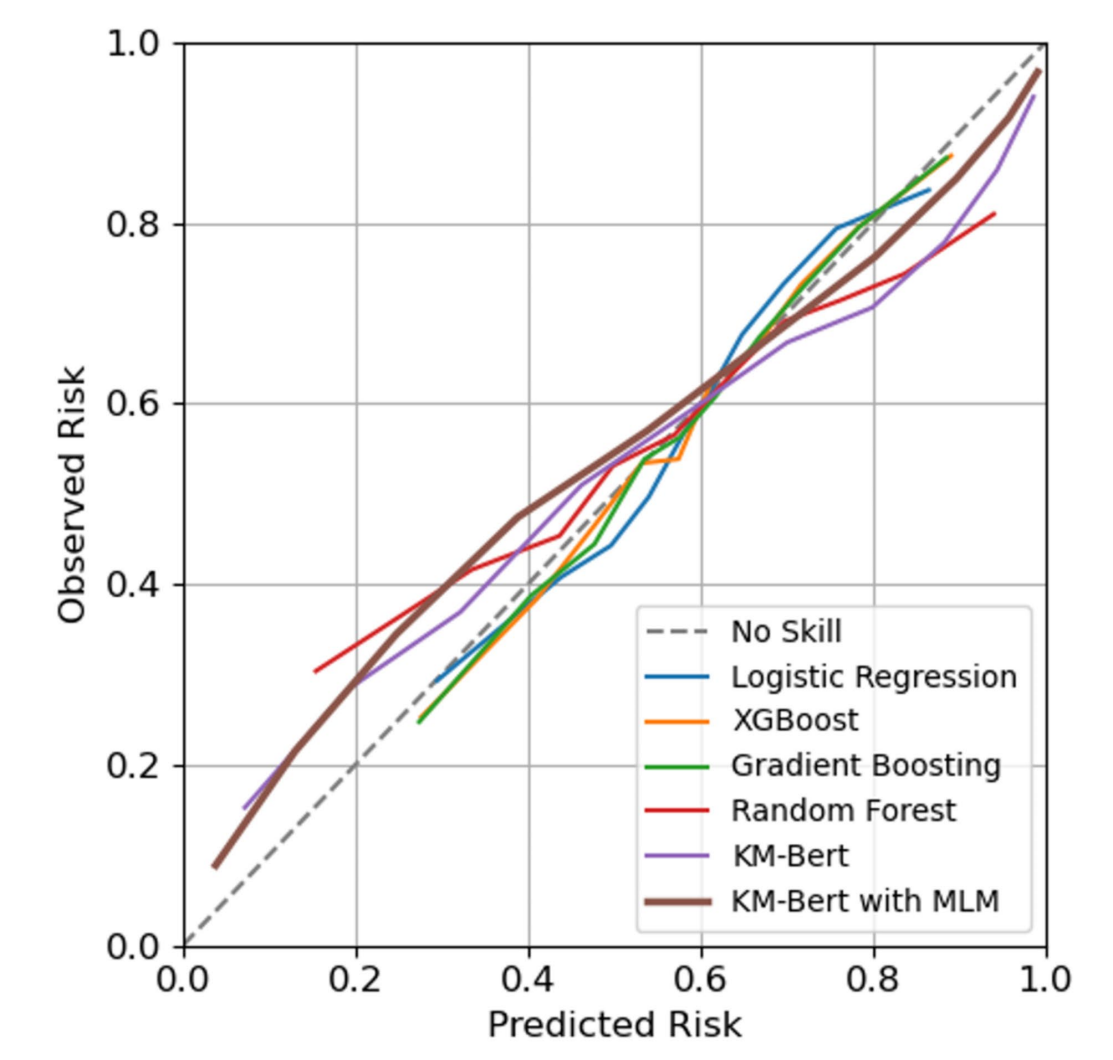
Calibration plots for natural language processing techniques and topic models. The observed risk compared to the predicted risk. A reference line indicates that the predicted risk and the observed emergency patient rate are exactly the same.

Calibration ensures that the predicted probabilities from a prediction model align with the actual observed frequencies. In addition to comparing Brier scores, we have included calibration curves (i.e., Q-Q plots) to check the quantile relationship between the predicted probability of each prediction model and the observed event rates. If the model is perfectly calibrated, the points on the Q-Q plot will fall along a 45-degree line, indicating that the predicted probabilities accurately reflect the true event frequencies. Figure 4 confirms that each model exhibited good prediction performance. However, we observed that the topic models with TF-IDF do not fully span the range from 0 to 1, indicating that these models’ calibration may be less reliable (might have a bias) when predicting probabilities close to 0 or 1.

### Rationalization

**Fig. 5:**
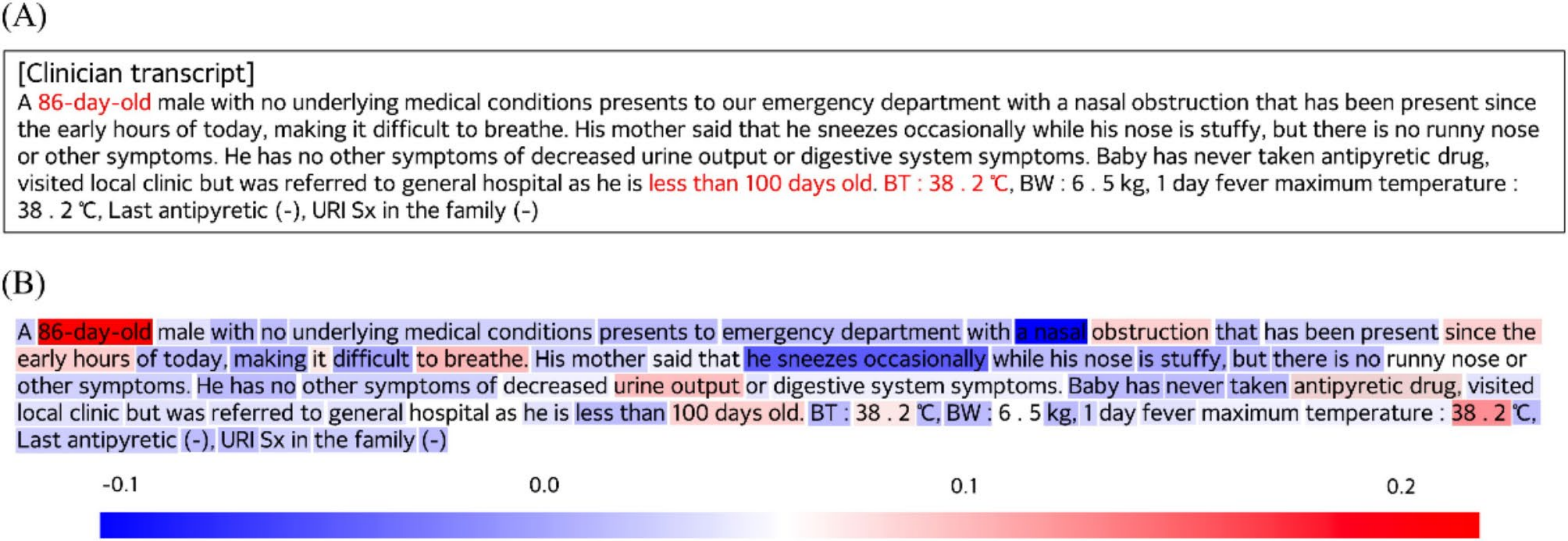
Comparison of passages marked as important by clinicians and those judged important by our DL-based prediction model to assess the emergency of pediatric patients. (A) Clinician transcript with important words marked in red by the clinician; the clinician marked the important words “86 days old,” “less than 100 days old,” and “temperature of 38.2 degrees” in red letters. (B) Clinician transcript with important words colored in red and unimportant words colored in blue by the DL-based prediction model.

We analyzed the results from an ML-based prediction model using TF-IDF. The TF-IDF scores of words extracted by this model are presented in Table A1 in the appendix, while the frequency of these words is visualized in Figure A1. For rationalization, we compared passages judged by an emergency specialist and those judged by our DL-based prediction model using the same clinician transcripts. The passages in which the specialist judged an emergency (red in Fig. 5A) and the passages in which our prediction model judged an emergency were measured similarly (red in Fig. 5B).

### Subgroup analysis with KTAS

**Fig. 6:**
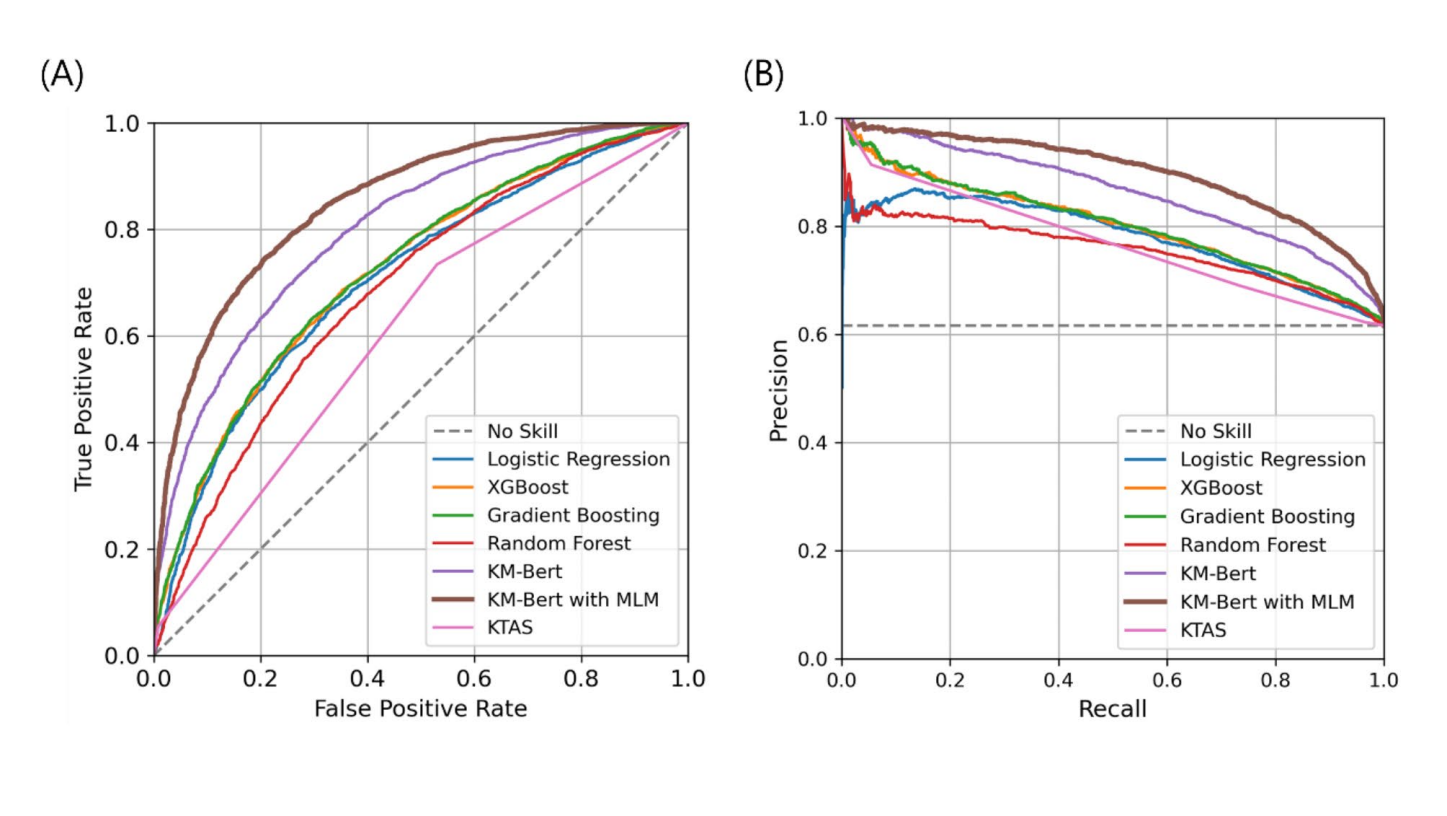
Prediction Ability of the conventional triage system (KTAS), and ML-based and DL-based NLP Techniques for Pediatric Emergency Prediction. (A) Receiver operating characteristic curves. (B) Precision-recall curve. The corresponding values of the area under the curve for each model are shown in Table 2. KTAS, Korean Triage and Acuity Scale; KM-BERT, Korean medical bidirectional encoder representations from transformers; MLM, masked language modeling; AUROC, area under the receiver operating characteristics; AUPRC, area under the precision-recall curve.

Because we measured KTAS scores in patients who visited the PED in 2016, we performed a subgroup analysis using the 47,968 cases in our dataset for which KTAS scores were recorded. In this analysis, KM-BERT with MLM achieved the highest AUROC of 0.849 ± 0.003, AUPRC of 0.896 ± 0.003, recall of 0.748 ± 0.004, precision of 0.842 ± 0.002, F1-score of 0.792 ± 0.003, and accuracy of 0.760 ± 0.003 and the lowest Brier score of 0.156 ± 0.002. KM-BERT also excelled, surpassing gradient boosting and KTAS with AUROC of 0.800 ± 0.003, AUPRC of 0.861 ± 0.003, recall of 0.748 ± 0.004, precision of 0.792 ± 0.003, F1-score of 0.769 ± 0.003, accuracy of 0.724 ± 0.003, and Brier score of 0.179 ± 0.002. Gradient boosting followed with AUROC of 0.726 ± 0.003, AUPRC of 0.802 ± 0.004, recall of 0.662 ± 0.004, precision of 0.757 ± 0.005, F1-score of 0.706 ± 0.004, accuracy of 0.662 ± 0.004, and Brier score of 0.202 ± 0.001, while KTAS trailed behind all models in each metric with AUROC of 0.6082, AUPRC of 0.6777, recall of 0.7324, precision of 0.6834, F1-score of 0.707, accuracy of 0.6281, and Brier of 0.2707 (Table 2; Fig. 6).

## Discussion

We assessed various models to predict emergencies in PEDs. The DL-based NLP techniques demonstrated superior reliability and prediction accuracy compared with the ML-based models using TF-IDF. In particular, KM-BERT with the MLM model exhibited exceptional performance across all metrics. Furthermore, rationalization confirmed that the emergency assessments by the experts were in close agreement with the results of the DL-based prediction model. Compared to those of the KTAS judgment scale, the DL-based prediction model’s predictions were the most accurate, underscoring the high reliability and precision of the fine-tuned model in analyzing pediatric emergencies.

In the medical field, TF-IDF has been widely utilized for various purposes, such as document classification, information retrieval, and patient data analysis. The strength of this model is its ability to easily transform unstructured text data into a structured format to extract meaningful patterns and insights^24,25^. Additionally, ML techniques have been used to predict hospitalization during ED triage. Several studies have shown that ML models can effectively predict hospitalization by combining patient history and triage information. These models were developed using various algorithms such as logistic regression, XGBoost, and deep neural networks (DNN)^26^. One of studies presented a DNN-based model that predicted PED admissions and outperformed existing methods by achieving an AUC of 0.892, demonstrating the importance of text data in improving prediction accuracy^27^. However, because TF-IDF simply analyzes text based on the frequency and importance of words, it has limitations in capturing the complex context or semantic nuances of the text.

Our findings are consistent with those of other studies in the field, highlighting the critical significance of DL-based NLP methods in current medical data analysis. The performance of the fine-tuned DL models was superior to that of the ML models using TF-IDF. DL-based NLP methods have played a significant role in achieving high accuracy in predicting hospital admissions and severe diseases. These models enhance predictive performance by integrating structured and unstructured text data and effectively identifying meaningful patterns and relationships across various data types^28^. According to this study, cutting-edge NLP techniques with DL have been successfully used to predict ED patient dispositions based on nursing triage records. These results indicate that DL-based NLP can effectively evaluate unstructured clinical text to predict patient outcomes in the ED and that paragraph vectors can provide the most accurate predictions^14^.

As shown in Fig. 5, we found that what ED clinicians recorded as important in assessing the patient’s emergency status was consistent with what the DL-based prediction model deemed important. The explanatory power of the DL-based prediction model can be used to identify the important factors when interviewing or examining emergency patients. In practice, inexperienced clinicians or pediatric emergency physicians may miss patients who are urgently ill despite having been interviewed in the ED, which can lead to a return visit to the ED. This can result in significant patient harm and inefficient use of emergency medical resources. DL-based prediction models can be used to train inexperienced clinicians and help them recognize patients in distress during patient encounters in a real-world ED setting.

Additionally, the urgency of pediatric patients presenting with PED is currently classified using various triage systems. However, as we found in a subgroup analysis in our study, traditional triage systems performed significantly worse than the DL-based prediction models at predicting emergency patients. DL-based prediction models can be used to accurately identify emergency patients so that aggressive treatment can be provided quickly. This is a desirable direction for improving clinical outcomes and ensuring patient safety.

Despite this, Word2Vec only considers neighboring words within a relatively small window, which limits its ability to capture the semantics of long sentences and the overall statistical information of the entire corpus. Although LSTMs are designed to handle long sequences, they still face challenges in learning relationships between distant words within a sentence due to the vanishing gradient problem. Furthermore, training large LSTM models is computationally infeasible because of their inherent sequential nature. These limitations make LSTMs less suitable for constructing large language models that can effectively learn meaningful semantics from extensive datasets, especially in the medical domain.

The prediction model in our study was built upon a more recent development in NLP called BERT^29^. BERT has gained considerable attention for overcoming the limitations of traditional DL approaches by adopting a transformer architecture as the basic building block^30^. In particular, BERT can provide significant performance improvements in comprehending natural languages by extracting semantically meaningful interactions in long sequences and constructing large language models with large datasets. Recently, BERT-based models have been widely utilized in the medical field^31–33^ after pretraining on medical corpora to bridge the gap between text used in the general domain and that in the medical domain. In this study, we adopted the pretrained KM-BERT^34^, a BERT-based model pretrained based on the Korean medical corpus (including medical textbooks, health information news, and medical research articles), as the backbone of our model to handle clinical transcripts primarily written in Korean.

This study has a few limitations. First, it was conducted using a retrospective cohort of clinician transcripts from a single tertiary hospital’s PED. Given a hospital’s tertiary status, it is believed that there is sufficient emergency care for severely ill patients. Second, transcripts were recorded by clinicians with varying degrees of skill and experience. However, DL-based prediction models are intended to comprehend and interpret the nuances of natural language, making them capable of processing and evaluating texts with diverse styles and levels of complexity. Thirdly, the pretrained KM-BERT, which was adopted as the backbone of our model, was trained based on the Korean medical corpus. While clinical records may contain some medical terms in English, the majority of crucial information, including chief complaints and symptoms, is documented in Korean. Therefore, a pre-trained model like KM-BERT, which is capable of effectively understanding both the Korean language and Korean-based medical terminology, was most appropriate for our research objectives. Nevertheless, we also recognize the importance of medical literature and terminology written in English. Recent advancements in models trained on English corpora, such as BioMistral^35^, further underscore the need to incorporate multilingual approaches in future research. Additionally, a significant limitation of study is the lack of external validation data, which may result in incorporation bias in our AUROC and AUPRC metrics and lead to potential overestimation of the model’s performance due to information leakage between the training and testing phases. Future studies should aim to validate these findings using external, independent datasets to confirm the generalizability and robustness of the model.

## Conclusion

Our findings highlight the increasing importance of advanced NLP techniques in the medical field, particularly during emergencies. The ability of these models to make precise and accurate forecasts can substantially benefit the management of healthcare resources, the improvement of patient care, and, ultimately, the overall efficiency of emergency medical services.

**Table 1:** Performance of pediatric emergency prediction using natural language processing techniques and topic models.

Variable	Logistic regression	XGBoost	Gradient boosting	Random forest	KM-BERT	KM-BERT with MLM^*^
AUROC	0.698 ± 0.002	0.714 ± 0.002	0.715 ± 0.002	0.680 ± 0.002	0.788 ± 0.002	**0.839 ± 0.001**
AUPRC	0.752 ± 0.002	0.776 ± 0.001	0.778 ± 0.001	0.735 ± 0.002	0.837 ± 0.002	**0.879 ± 0.001**
Recall	0.629 ± 0.003	0.618 ± 0.002	0.626 ± 0.003	0.625 ± 0.002	0.719 ± 0.002	**0.724 ± 0.002**
Precision	0.728 ± 0.002	0.741 ± 0.001	0.737 ± 0.001	0.716 ± 0.002	0.775 ± 0.002	**0.829 ± 0.001**
F1-score	0.675 ± 0.002	0.674 ± 0.002	0.677 ± 0.001	0.667 ± 0.001	0.746 ± 0.002	**0.773 ± 0.001**
Accuracy	0.643 ± 0.002	0.648 ± 0.002	0.649 ± 0.001	0.633 ± 0.002	0.712 ± 0.002	**0.749 ± 0.001**
Brier	0.215 ± 0.000	0.209 ± 0.000	0.209 ± 0.000	0.225 ± 0.001	0.188 ± 0.001	**0.164 ± 0.001**

KM-BERT, Korean medical bidirectional encoder representations from transformers; MLM, masked language modeling; AUROC, area under the receiver operating characteristics; AUPRC, area under the precision-recall curve.

Bold values indicate the best-performing model across all metrics.; ^*^Indicates overall best-performing model.

**Table 2:** Performance of pediatric emergency prediction using natural language processing techniques and topic models compared to KTAS.

Variable	KTAS	Logistic regression	XGBoost	Gradient boosting	Random forest	KM-BERT	KM-BERT with MLM^*^
AUROC	0.610 ± 0.002	0.709 ± 0.003	0.723 ± 0.003	0.726 ± 0.003	0.691 ± 0.003	0.800 ± 0.003	**0.849 ± 0.003**
AUPRC	0.679 ± 0.003	0.780 ± 0.003	0.801 ± 0.003	0.802 ± 0.004	0.761 ± 0.004	0.861 ± 0.003	**0.896 ± 0.003**
Recall	0.733 ± 0.003	0.655 ± 0.004	0.654 ± 0.003	0.662 ± 0.004	0.650 ± 0.003	0.748 ± 0.004^†^	**0.748 ± 0.004** ^†^
Precision	0.685 ± 0.003	0.754 ± 0.004	0.758 ± 0.004	0.757 ± 0.005	0.738 ± 0.004	0.792 ± 0.003	**0.842 ± 0.002**
F1-score	0.708 ± 0.002	0.701 ± 0.004	0.702 ± 0.003	0.706 ± 0.004	0.691 ± 0.003	0.769 ± 0.003	**0.792 ± 0.003**
Accuracy	0.628 ± 0.002	0.657 ± 0.003	0.660 ± 0.003	0.662 ± 0.004	0.644 ± 0.003	0.724 ± 0.003	**0.760 ± 0.003**
Brier	0.271 ± 0.001	0.209 ± 0.001	0.203 ± 0.001	0.202 ± 0.001	0.216 ± 0.001	0.179 ± 0.002	**0.156 ± 0.002**

KTAS, Korean Triage and Acuity Scale; KM-BERT, Korean medical bidirectional encoder representations from transformers; MLM, masked language modeling; AUROC, area under the receiver operating characteristics; AUPRC, area under the precision-recall curve.

Bold values indicate the best-performing model across metrics.

^*^Indicates overall best-performing model; ^†^Indicates tied best performance for Recall.

## Electronic supplementary material

Below is the link to the electronic supplementary material.

Supplementary Material 1

Supplementary Material 2

## Data Availability

Deidentified data supporting the findings of this study are available from the corresponding authors upon request.
