# Supplementary material for: A pediatric emergency prediction model using natural language process in the pediatric emergency department
Source: Sci Rep. 2025 Jan 28;15:3574. doi: 10.1038/s41598-025-87161-x (PMC11775304; doi:10.1038/s41598-025-87161-x)
Supplement: Supplementary file 1 — Supplementary Material 1 [file 41598_2025_87161_MOESM1_ESM.docx]

**Appendix Figure**

**
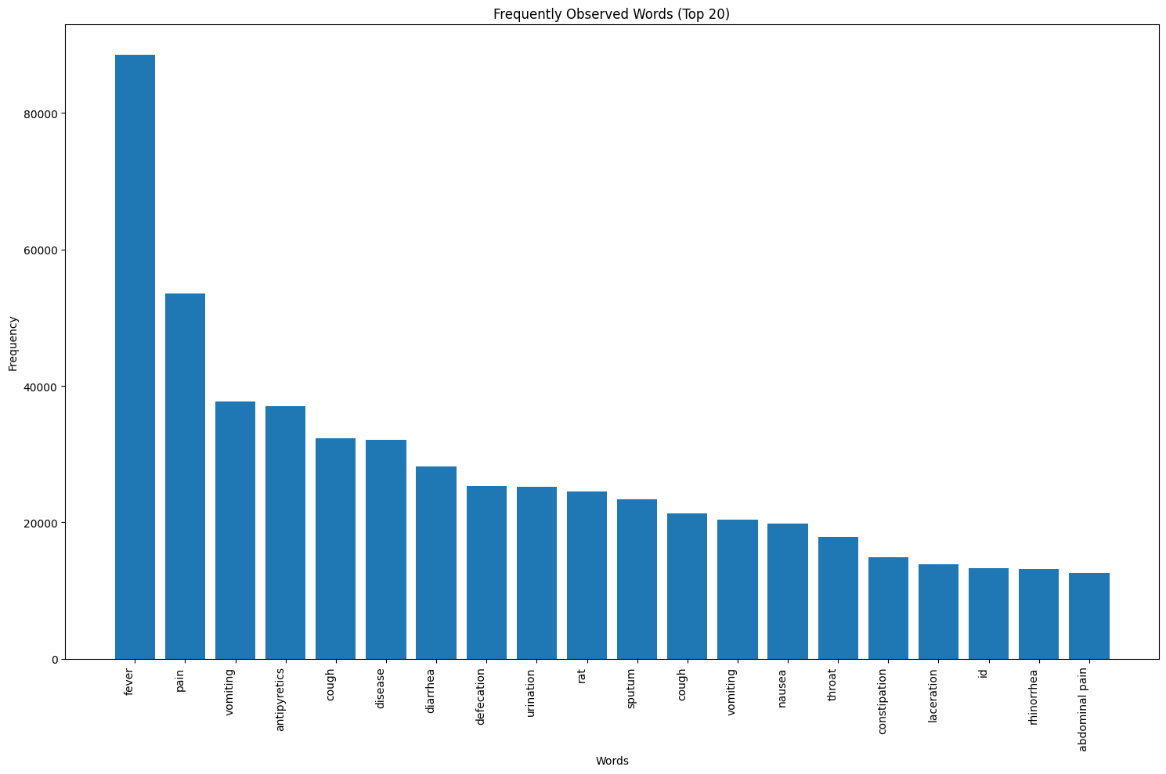
**

Figure A1. Frequency of words extracted by machine learning-based prediction model

**Appendix Table**

Table A1. TF-IDF score of words used in ML-based prediction model

| **Ranking** | **Word** | **TF-IDF score** |
| --- | --- | --- |
| 1 | eye* | 0.333 |
| 2 | vomiting | 0.311 |
| 3 | test | 0.31 |
| 4 | corona* | 0.288 |
| 5 | corona* | 0.288 |
| 6 | id | 0.263 |
| 7 | hemorrhage | 0.251 |
| 8 | rhinorrhea* | 0.247 |
| 9 | rhinorrhea* | 0.242 |
| 10 | eye* | 0.24 |
| 11 | laceration | 0.237 |
| 12 | Ischemia* | 0.232 |
| 13 | corona | 0.225 |
| 14 | skin | 0.225 |
| 15 | fever | 0.225 |
| 16 | cough | 0.215 |
| 17 | urticaria | 0.214 |
| 18 | Ischemia* | 0.212 |
| 19 | otalgia | 0.208 |
| 20 | medication | 0.207 |
| ^*^If the ML-based prediction model determines that a particular word is important in multiple texts, the same word appears multiple times in the ranking.  TF-IDF: Term frequency-inverse document frequency, ML: Machine learning. | | |
