# Supplementary material for: A pediatric emergency prediction model using natural language process in the pediatric emergency department
Source: Sci Rep. 2025 Jan 28;15:3574. doi: 10.1038/s41598-025-87161-x (PMC11775304; doi:10.1038/s41598-025-87161-x)
Supplement: Supplementary file 2 — Supplementary Material 2 [file 41598_2025_87161_MOESM2_ESM.docx]

Supplementary Figure 1. Distribution of emergency cases by intervention criteria and disposition
